# Feasibility of middle colic artery as a landmark for superior mesenteric artery – first approach in laparoscopic pancreatoduodenectomy: a prospective study

**DOI:** 10.1186/s12957-024-03416-3

**Published:** 2024-05-28

**Authors:** Ham Hoi Nguyen, Thanh Khiem Nguyen, Hong Son Trinh, Hai Dang Do, Tuan Hiep Luong, Hoan My Pham, Van Duy Le, Van Minh Do, Pisey Chantha, Hong Quang Pham, Dang Vung Nguyen

**Affiliations:** 1 Hanoi Medical University, Hanoi, Vietnam; 2 Center of Gastrointestinal and Hepato-Pancreato-Biliary Surgery, Bach Mai Hospital, Hanoi, Vietnam; 3 Deparment of Oncology, Viet Duc University Hospital, Hanoi, Vietnam; 4 Organ Transplantation Center, Viet Duc University Hospital, Hanoi, Vietnam; 5 VinUniversity, Hanoi, Vietnam; 6 Thai Binh University of Medicine and Pharmacy, Thai Binh, Vietnam; 7 Department of Gastrointestinal and Hepato-Pancreato-Biliary Surgery, Bach Mai Hospital, Hanoi, Vietnam

**Keywords:** Laparoscopic pancreaticoduodenectomy, Artery-first approach, Middle colic artery

## Abstract

**Background:**

SMA-first approach in pancreatoduodenectomy (PD) has been widely applied in open surgery as well as laparoscopy. Finding the superior mesenteric artery (SMA), inferior pancreatoduodenal artery (IPDA), first jejunal artery (J1A) has become a great challenge in laparoscopic PD (LPD). Meanwhile, exposing the midde colic artery (MCA) might be a feasible approach to determine SMA, IPDA, and J1A. Our study aims to find the anatomical correlation between MCA and SMA, IPDA, J1A, especially in SMA-first approach LPD from the left.

**Methods:**

Uncontrolled clinical trial with 33 patients undergoing LPD had preoperative contrast abdominal CT scan to analyze the anatomical relevance between MCA and SMA, J1A, IPDA. The operation was performed starting with exposing MCA in advance to find SMA, J1A and IPDA. The data was analyzed by SPSS 25.0.

**Results:**

90.9% of MCA started at 12–3 o’clock from SMA, the mean distance from the SMA root to the MCA and J1A was 56.4 mm and 37.4 mm, respectively. The distance between SMA and J1A was 19 mm. 72.7% J1A started at 9–12 o’clock, 69.7% J1A and IPDA had a common trunk. 78.8% IPDA started at 3–6 o’clock. 100% of the cases had J1A controlled intraoperatively, 81.8% for IPDA when approached from the left, 3% had MCA injury. The mean time to approach from the left was 98 min, median blood loss was 100 ml.

**Conclusion:**

Exposing MCA first helps determine SMA, J1A and IPDA safely, efficiently and faciliates SMA-first approach LPD from the left and complete dissection of the mesopancreas and lymph nodes.

## Introduction

SMA-first approach in PD has proved its efficacy in minimizing blood loss, resecting completely mesopancreas, achieving R0 resection, and prolonging survival time [1, 2]. LPD with SMA-first approach has been increasingly published in specialized centers [3]. However, this method was still a challenge due to demanding technical skills in dissection and exposure of SMA, IPDA and J1A [4]. Left posterior approach, which means approach firstly from the left-posterior side of SMA of the patient’s body, has been proven to be beneficial: first approach to SMA and early control of IPDA and J1A [5]. We discover that initiating the approach from the left side of the superior mesenteric artery (SMA) offers numerous benefits. These include simplified pulse regulation owing to direct SMA access. Additionally, in the majority of cases, both the inferior pancreaticoduodenal artery (IPDA) and the 1st jejunal artery (F1A) stem from the left side of the SMA. Furthermore, lymph nodes and connective tissue situated along the left-sided SMA can be readily excised. During dissection, we discovered that MCA could be a helpful landmark in the search for SMA, IPDA and J1A, especially in complicated cases with pancreatitis or obesity. Therefore, we examined the anatomical relationship between MCA and other fundamental vessels in LPD based on preoperative imaging study to apply this landmark in practical scenario of the surgery.

## Methods

After an Institution Review Board (IRB) approval, the patient was included if they had periampullary tumor at the resectable stage, indicated for LPD with given protocol with SMA-first approach from the left. The patients should have a good physical status with no contraindications for laparoscopy and normal coagulation function. Preoperative biliary drainage might be indicated if serum bilirubin > 250mmol/L. Exclusion criteria: Vascular invasion suspected, withdrawal from research.

**Fig. 1:**
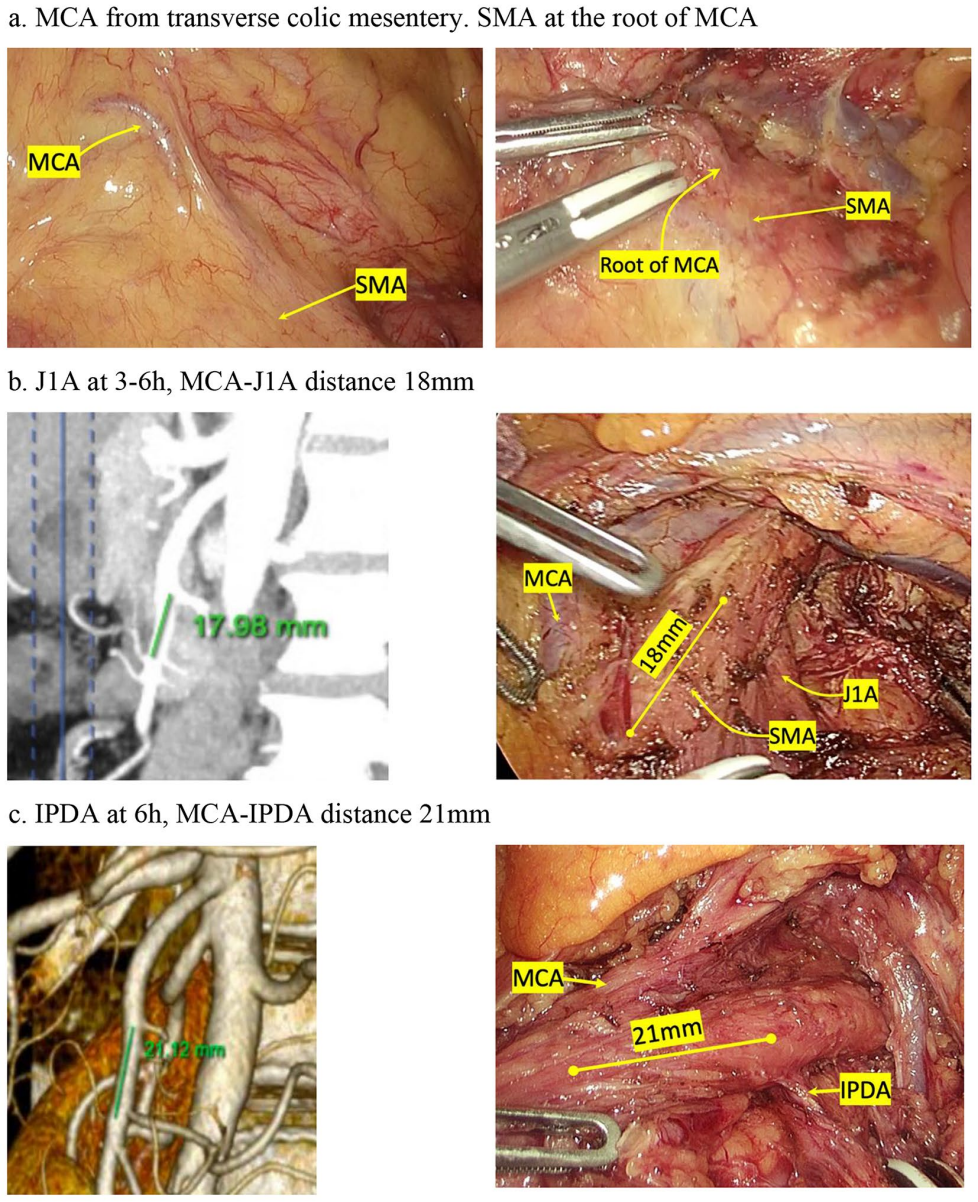
Relationship between MCA, J1A, IPDA and SMA. (J1A: First jejunal artery; IPDA: Inferior pancreatoduodenal artery; MCA: middle colic artery; SMA: Superior mesenteric artery)

Our study was an uncontrolled clinical trial. Totally, 33 patients who agreed to participate with the above-mentioned criteria from January 2021 to August 2023 were included. All of our patients went through a thorough workup, including a contrast CT scan to evaluate anatomical variants of vessels and plan for vascular approach and control. The imaging was made with Maximum Intensity Projection by an experienced radiologist (See Fig. 1).

## Surgical protocol

All surgeons included in our study were highly experienced with at least 20 cases of LPD and 50 cases of open PD. All cases in the study were performed according to a unified surgical procedure that have been published in our previous technical article with detailed step-by-step video [6].

- Place the patient supine, two arms abducted, abduct both legs 60 degrees. Set 5 abdominal trocars. After exploration, approach to the SMA from the left side: The assistant stretches out transverse colon mesentery and first jejunal loop, surgeon ligates Treitz ligament and mobilizes IMV. In case when IMV drained into SMV, ligate the IMV.
- MCA could be exposed right below transverse colic mesentery. From the MCA, approach to the anterior surface of SMA via posterior peritoneum. After determining the SMA axis, dissect from up to down, from the anterior section to the left posterior side, as the anterior side is considered an avascular region. Expose and ligate the J1A if it is separated or the common trunk with IPDA is short. The first jejunum was then cut.
- The IPDA is determined by the relative distance and direction with the MCA. If IPDA stems independently from the plane cross 2/3 of the diameter of the SMA left border, we will ligate IPDA. If IPDA/PIPDA stems from the right border, the artery will be preserved to be ligated later. Continuing SMA dissection to the right side till its root (above the left renal vein), perform the lymphadenectomy around the SMA. Mobilize the structure from the anterior surface of inferior vena cava and abdominal aorta.
- Approach the SMV, ligate the Henle trunk, split the pancreatic neck from the SMV. Cut the gastric antrum, dissect the celiac trunk lymph node, hepatic pedicle, ligate the gastroduodenal artery. Split the pancreatic neck, finalize the mobilization around the SMA. The gallbladder and common bile duct was lastly removed.
- Do the anastomosis: Either total laparoscopic manual anastomosis or mini-laparotomic manual anastomosis via a small abdominal incision (approximately 7 cm) was conducted in all cases. In each instance, a double-layer pancreatoenteric end-to-side anastomosis, specifically the modified Blumgart anastomosis, was executed. The biliary-enteric anastomosis was created in a single layer, positioned distally to the pancreatoenteric anastomosis by approximately 10 cm. Lastly, an antecolic gastro-jejunostomy was performed using a distal loop of jejunum (approximately 60 cm distal to both the biliary-enteric anastomosis) and gastric.
- Place the drainage and do the abdominal closure.

### Data collection and analysis

- Patient demographics: Demographic characteristics (sex, age, height, weight, BMI, medical history),
- MCA, J1A and IPDA anatomical parameters:
  - MCA: Distance from MCA to SMA root (mm), correlation between MCA position to SMA circumference (classified into 4 quadrants), and types of MCA based on Sobal classification [7]:
    - Type I: Right colic artery (RCA), MCA and ileocolic artery (ICA) originate independently.
    - Type IIa: Common trunk between RCA and MCA.
    - Type IIb: Common trunk between RCA and ICA.
    - Type IIc: MCA and LCA in one.
    - Type III: MCA from celiac trunk.
    - Type IV: Others.
  - J1A: Distance from J1A to SMA root (mm), J1A position, correlation between J1A and IPDA (or PIPDA).
  - IPDA (or PIPDA) on the left, other anatomical variations (if presented).
- Techniques-related data: time for SMA approach, total operative time, J1A ligation, IPDA ligation from the left, peri-SMA nerve plexus preservation, extended lymphadenectomy, intraoperative complications, blood loss and blood transfusion; Conversion to open surgery, total number of lymph nodes, left-side SMA lymph nodes, mesopancreas metastasis.
- Postoperative complications, length of hospital stay, in-hospital mortality were collected. Histopathological data: Number of lymph nodes retrieved, number of metastatic lymph nodes, metastatic status of SMA left-sided LNs and resected margins status, which was defined as follows: no cancer cells are identified microscopically at any of the resected margins (R0), microscopic tumor present at any margins or within 1 mm of a transection or circumferential margin according to the Royal College of Pathologists guidelines (R1), and gross residual tumor as determined by the surgeon intraoperatively [8].

### Statistical analysis

Categorical variables were presented as a percentage of each value. Discrete variables were numeric variables that had a countable number of values between any two values. Continuous variables were expressed as mean (or median if without normal distribution) with range. Continuous variables were analyzed by the Wilcoxon rank sum test. Categorical variables were analyzed using the chi-square test or Fisher’s exact test. Statistical analysis was performed using SPSS for Windows, version 25.0 (SPSS Inc., Chicago, IL, USA).

## Results

There were total 33 patients in our study. The mean age was 58.7 ± 9.1 years (range 35–70). Mean BMI was 20.9 ± 2,0 (16.8–25) (See Table 1).

**Table 1:** Patient demographics

Characteristics	Results
Age (Mean ± SD, min-max)	58.7 ± 9.1 (35–70)
- 30–50	6 (18.2)
- 51–70	27 (81.8)
Sex	
- Male	16 (48.5)
- Female	17 (51.5)
Medical history	
- Alcohol	7 (21.2)
- Chronic liver diseases	1 (3.0)
- Chronic pancreatitis	1(3.0)
BMI (Mean ± SD, min-max)	20.9 ± 2,0 (16.8–25)
- < 18	2 (6.1)
- 18–25	30 (90.9)
- ≥ 25	1 (3.0)

### Anatomical characteristics of MCA

In our study, 100% of the patients had independent MCA roots. The mean distance from MCA to SMA root was 56.4 mm. Most of the patients’ MCA (90.9%) originates from the upper right quadrant of the SMA, and only 3% of the cases had MCA on the left side.

### Anatomical characteristics of J1A

The mean distance from J1A to the SMA root was 37.4 mm. Most of the cases (72.7%) had J1A originating from 3 to 6 o’clock of the SMA. In 69.7% of our cases, J1A and IPDA shared a common trunk. The mean distance from J1A to the MCA root was 19 mm.

We also recorded other vascular variations, including 3 (9.1%) cases with aberrant RHA, 4 (12.1%) cases with inferior pancreatic artery, 1 (3%) case with left colic artery stemming SMA, 10 (30.3%) IMV draining into SMV.

The mean time for the left SMA-approach in our study was 98 min. The mean total operative time was 433 min. The median total blood loss was 100 mL, ranging from 50 to 1500 mL. Only 1 case had uncontrollable blood loss due to transverse colon mesentery tear, which required intraoperative blood transfusion, and finally, conversion to open surgery.

In all of the cases, we succeeded in ligating J1A, preserving peri-SMA neural plexus and performing extended lymphadenectomy. We ligated IPDA from the left in 27 (81.8%) cases.

During the post-operative period, we recorded 4 cases with over Grade II complications, including 1 case suffered bleeding from SMV branch injury, requiring reoperation. 1 case with bleeding from the inferior epigastric branch on trocar placement, successfully embolized, 1 case of self-controlled secondary bleeding after grade B pancreatic fistula, and 1 case anastomotic bleeding, treated conservatively with blood transfusion. The mean length of stay at the hospital was 14 days, ranging from 8 to 25 days.

Pathology results revealed mean number of collected lymph nodes was 33, with 7.3 collected to the left of the SMA due to extended lymphadenectomy. R0 resection was achieved in 31 (94%) cases.

## Discussion

### Anatomical variations of MCA

Anatomical variations cause the most challenge in vascular dissection. Our study showed 39.4% of the cases with J1V anterior to SMA, 30.3% with IMV draining into SMV, 12.1% had inferior pancreatic artery (IPA), 9.1% right hepatic artery (RHA) stemming from SMA and 1% had left colic artery (LCA). Sobal et al. also reported an incidence of 2% of LCA stemming from SMA instead of from inferior mesenteric artery [7]. RHA must be preserved while J1V, IMV, IPA, LCA could be ligated without risk of ischemia. In the case of aberrant RHA, dissection posteriorly to the SMA could be challenging due to a high risk of RHA injury.

Although many authors have described different approaches in open surgery, few authors reported a standard protocol for minimally invasive surgery, especially the left posterior approach [5, 9, 10]. In SMA-first approach, it is important to first identify the SMA, IPDA, and J1A. Studies show that the majority of IPDA originates in the same trunk as the J1A on the left side of the SMA. Left posterior approach has the following advantages: direct access to the SMA, easy control of the IPDA and J1A due to anatomical correlation. Furthermore, according to Nagakawa, the anterior left of the SMA is an avascular space, which facilitates dissection [11]. 

### Anatomical variations of MCA in approach to SMA, J1A, IPDA

Basically, RCA, MCA and ICA originate independently from SMA. In Sobal’s study, it is the most common circumstances (82%), and 7 (14%) cases with common MCA and RCA trunk [7]. Soneland et al. showed an incidence of type 2a of 26.7% [12]. This was similar to our results, showing the majority of type 1 and a lower incidence of type 2a.

**Fig. 2:**
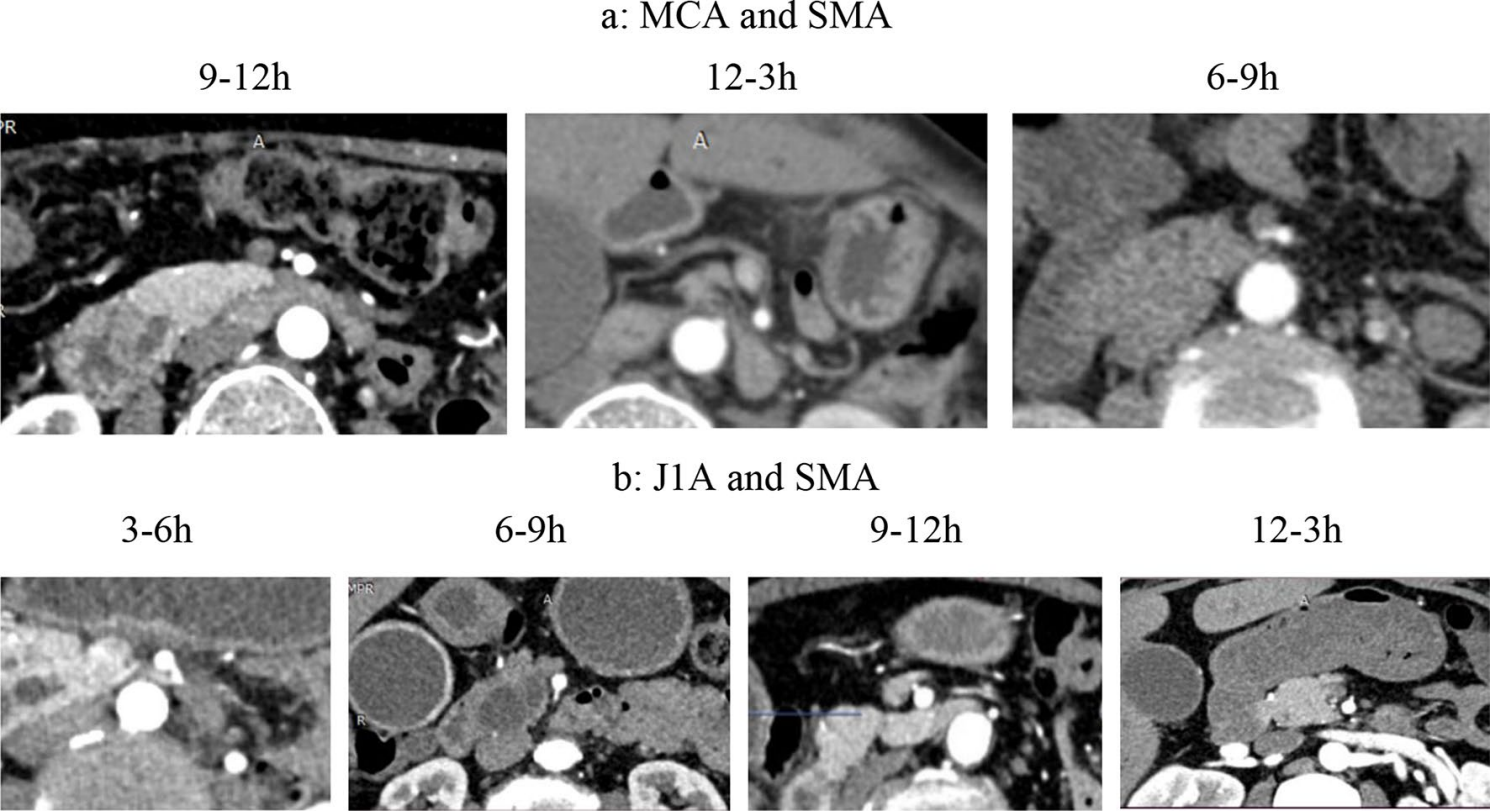
Position of MCA, J1A in correlation with SMA circumference. (J1A: First jejunal artery; IPDA: Inferior pancreatoduodenal artery; MCA: middle colic artery; SMA: Superior mesenteric artery)

We divided the MCA origin from SMA position into 4 regions clockwise: 12–3, 3–6, 6–9, and 9–12 o’ clock. Our study showed 90.9% of MCA stemming at 9–12 o’clock of the SMA (Fig. 2).

The mean distance between SMA and MCA root was 56 mm, comparable to the inferior border of the pancreas and MCA could be easily found when the colic mesentery and the first jejunal loop were stretched out. This result was similar to that reported by Horiguchi (54 mm) [13]. The author’s study also showed that the distance from MCA to IPDA was significantly shorter than from IPDA to SMA root, and therefore, it was feasible and safe for MCA to be a better landmark than the SMA root to identify IPDA. Horiguchi also highlighted the importance of preoperative measurement of these vessels on imaging study for a better operative plan to find IPDA based on the MCA [13]. 

**Fig. 3:**
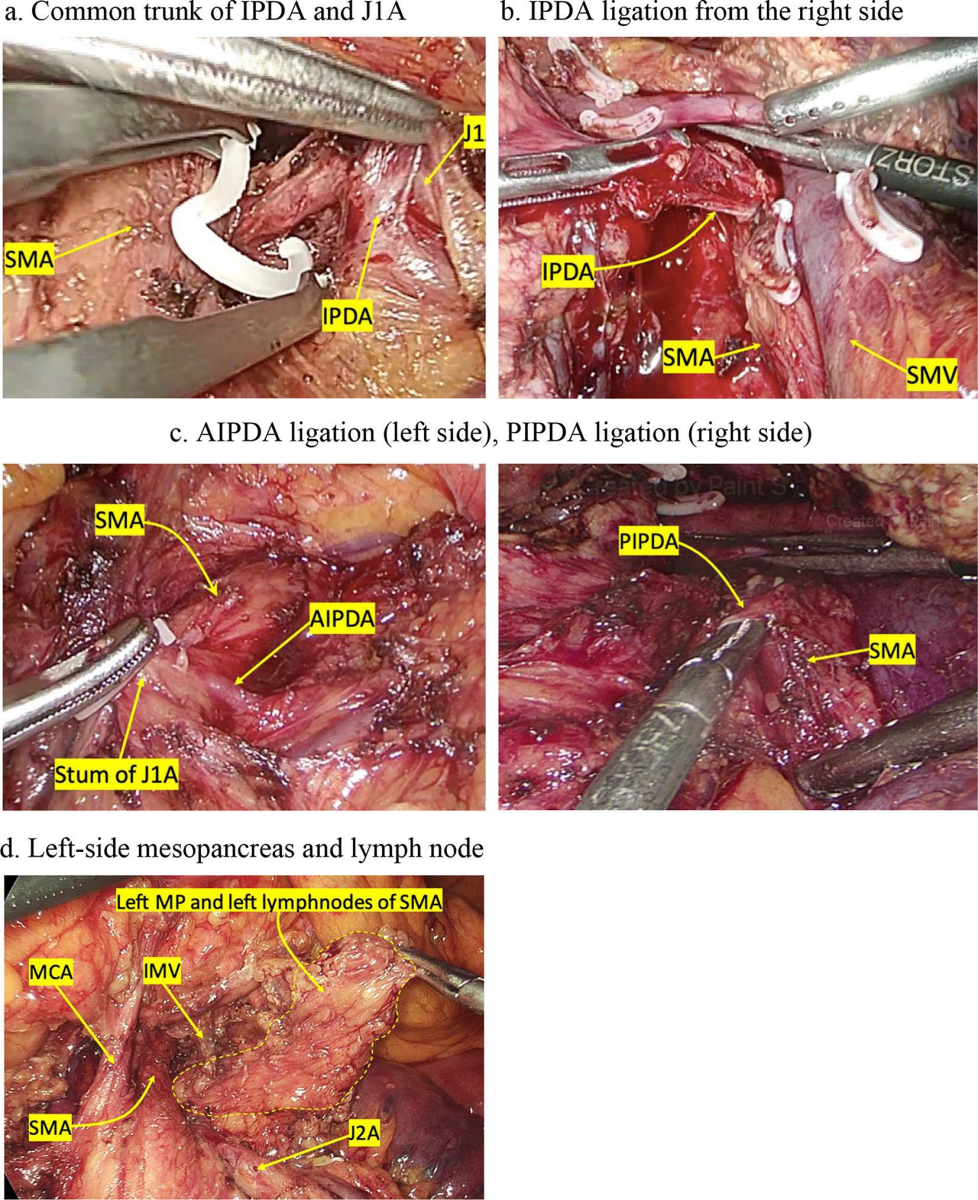
Ligation of IPDA, anterior/posterior IPDA (AIPDA/PIPDA), left-side lymph node. (AIPDA: Anterior inferior pancreatoduodenal artery; J1,2 A: First, second jejunal artery, IMV: inferior mesentery vein; IPDA: Inferior pancreatoduodenal artery; MCA: middle colic artery; PIPDA: posterior inferior pancreatoduodenal artery; SMA: Superior mesenteric artery; SMV: Superior mesenteric vein)

MCA frequently stemmed independently at 6–9 o’clock from the SMA. Therefore, MCA should be considered as a helpful and constant landmark in identifying SMA in artery-first approach. In other words, SMA was identified when the MCA was determined (Fig. 3).

Analysis of the relationship between MCA and J1A showed that: J1A originated at 37.4 mm from the origin of SMA, above MCA and the average distance between MCA and J1A was 19 mm (the smallest distance measured on MSCT was 2 mm). Evaluation of position of J1A root compared to SMA showed that 72.7% were at 3–6 o’clock, 21.2% were at 6–9 o’clock (Table 2). Thus, to identify J1A, we can find and follow upward the J1A by 19 mm in an upward direction of about 19 mm, deviating to the left edge of SMA at about the 3–6 o’clock position.

**Table 2:** Anatomical characteristics of MCA, IPDA and J1A

Characteristics	Results
Independent MCA root	100%
Distance from MCA to SMA root	56.4±9.8 mm (30–77)
MCA position in correlation with SMA - 9–12 o’clock	90.9%
- 12 − 3 o’clock	6.1%
- 6–9 o’clock	3%
Distance from J1A to SMA root	37.4 ± 8.4mm (22-50.9)
J1A position in correlation with SMA	
- 3–6 o’clock:	72.7%
- 6–9 o’clock	21.2%
- 12 − 3 o’clock	3%
- 9–12 o’clock	3%
Distance from J1A to MCA root	19 ± 7.8 mm (2–33)
Correlation between J1A and IPDA (PIPDA)	
- Common trunk	69.7%
- Independence	30.3%
IPDA common trunk present	81.7%
IPDA or PIPDA on the left	78.8%
**Other anatomical variations**	
Aberrant RHA from SMA	3 (9.1%)
Inferior pancreatic artery	4 (12.1%)
Left colic artery from SMA	1 (3%)
J1V anterior to SMA	13 (39.4%)
IMV drained into SMV	10 (30.3%)

In addition, J1A was closely related to IPDA and PIPDA. Our research results showed that 69.7% of IPDA or PIPDA originated from J1A. This was consistent with other studies like Yoshiya Ishikawa (66%) and Yasunari Kawabata (74.3%) [14, 15]. Meanwhile, Murakami reported a common trunk of IPDA and J1A in 58.9% of cases, independent trunk of IPDA from SMA in 24.2%, and 16.9% with IPDA stemming from both sites, 70.6% of which had IPDA (PIPDA) stemming from the left side of the SMA [16]. Our study showed similar results with 78.8% of the cases with common trunk of IPDA and PIPDA branches originating on the left side of the SMA. Our study illustrated a close relationship between J1A and IPDA, and therefore, identifying J1A allowed IPDA to be controlled. Though the race and ethnicity are different, their relationship is similar (See Table 3).

**Table 3:** Intraoperative techniques and outcomes

Characteristics	Results
Time for left SMA-approach (minutes)	98 ± 27.9 (60–153)
Total operative time (minutes)	433.3 ± 80.2 (250-600)
Blood loss during SMA approach (mL)	84.2 ± 52.8 (30-250)
Total blood loss (mL)	143 ± 252 (50–1500)
Intraoperative blood transfusion (case)	1 (3%)
Conversion to open surgery (cases)	1 (3%)
J1A ligation	33 (100%)
IPDA ligation from the left (cases)	27 (81.8%)
**Early outcomes**	
Post-operative complications (Clavien-Dindo)	
- II	1 (3%)
- IIIa	2 (6%)
- IIIb	1 (3%)
Length of stay (days)	14.3 ± 4 (8–25)
**Oncological outcomes**	
Total number of collected lymph nodes	33 ± 12.7 (16-74)
Site	
- Pancreatic head	8 (24,2)
- Distal CBD	5 (15,2)
- Vater	20 (60,6)
Number of lymph nodes to the left of SMA	7.3 ± 5.9 (3-30)
Patients with positive lymph nodes	18 (54.5%)
Patients with positive left-sided lymph nodes	5 (15.2%)
Patients with mesopancreas metastasis	8 (24.2%)
R0 resection rate*	31 (94%)

*One case of pancreatic parenchymal resection margin and one case of mesopancreas resection margin

### Intraoperative and postoperative complications

In our study, there is one case of intraoperative MCA injury during vascular dissection. Fortunately, the MCA could be safely ligated without causing colon ischemia. One case required conversion to open surgery due to hemodynamic instability caused by late recognition of bleeding mesentery. This is also a point to pay attention to when using the middle colon landmark, approaching from the left side and dissection of lymph nodes. The incidence of comorbidity in our study was equivalent to others performing left approach, and was not higher than those performing other SMA-first approach. Other publication show higher open conversion rate, such as Treeoongckaruna (17.7%), Boggi (9.1%), Feng Tian (16.7%) [17–19]. The median total blood loss was 100 mL. Our results were lower than that reported by Feng Tian (300 ml), and equivalent to Sameer’s study (110–350 ml) [19, 20]. Chen et al. compared 2 groups of 89 patients undergoing either open or laparoscopic PD. Total laparoscopic surgery required longer operative time than open surgery, but less blood loss and blood transfusion [21]. 

Our study implied that left approach did not increase complications in LPD. Boggi et al. found complication rates ranging from 18.1 to 64.2%, with an average of 41.2%. The mortality rate varied from 0 to 7.1% [18]. Our study found that the rate of complications at Clavien Dindo classification grade II or higher accounted for 12.1%. Chen et al. found that postoperative complications in the laparoscopic group were lower in both incidence and severity but not statistically significant [21]. None of our patients had DGE, bile leakage or bowel anastomosis leak.

Every types of SMA first approach has advantages and disadvantages, which has summarized by P. Sanjay et al. [22]. Our left-posterior first approach, with application of MCA as a landmark, offers several advantages over the posterior approach in terms of anatomy: This method provides direct access from the left side, targeting the free edge of the artery, with the RCA and MCA serving as landmarks. The colonic artery is visible without dissection due to the thin connective tissue in this region. The area between the SMA and SMV, as well as the splenic vein and the posterior retro-pancreatic tissue, consists of loose connective tissue, facilitating easy separation during surgery. Through the use of laparoscopic instruments and lenses, the posterior retro-pancreatic tissue can be easily and clearly observed. Furthermore, the anterior surface of the SMA, from the origin of the MCA and RCA to the origin of the SMA, is an avascular space, simplifying surgical navigation. Moreover, the posterior aspect of the SMV and the splenic vein is devoid of branches, which streamlines the dissection process. Last but not least, the majority of the IPDA and F1A originate from the left half of the SMA, aiding in precise anatomical identification and surgical intervention. Our study showed a feasible approach in Whipple’s procedure by using MCA as a landmark for SMA. Further randomized trials with larger sample sizes and longer follow-up should be conducted to prove their effectiveness.

## Conclusion

Exposing MCA first helps determine SMA, J1A and IPDA safely, efficiently and faciliates SMA-first approach LPD from the left and complete dissection of mesopancreas and lymph nodes to the left of the SMA. This study was conducted on a small group of patients at a single center.

## Data Availability

No datasets were generated or analysed during the current study.

## Abbreviations

AIPDA: Anterior inferior pancreatoduodenal artery
J1A: First jejunal artery
J1V: First jejunal vein
ICA: inferior colic artery
IMV: inferior mesentery vein
IPA: inferior pancreatic artery
IPDA: Inferior pancreatoduodenal artery
IPDV: Inferior pancreatoduodenal vein
LCA: left colic artery
LPD: Laparoscopic pancreaticoduodenectomy
MCA: middle colic artery
PD: pancreaticoduodenectomy
PIPDA: posterior inferior pancreatoduodenal artery
RCA: right colic artery
RHA: right hepatic artery
SMA: Superior mesenteric artery
SMV: Superior mesenteric vein
